# Evolutionary adaptations of *Pseudomonas aeruginosa* biofilms to ciprofloxacin and antioxidant co-treatment in synthetic sputum medium

**DOI:** 10.1128/spectrum.03149-25

**Published:** 2026-02-12

**Authors:** Doaa Higazy, Fauve Vergauwe, Tom Coenye, Oana Ciofu

**Affiliations:** 1 Department of Immunology and Microbiology, Costerton Biofilm Center, University of Copenhagen 4321, Copenhagen, Denmark; 2 Department of Microbiology, Faculty of Agriculture, Cairo University 63526, Giza, Egypt; 3 Laboratory of Pharmaceutical Microbiology, Ghent University 26656, Ghent, Belgium; Universita degli Studi Roma Tre Dipartimento di Scienze, Roma, Italy

**Keywords:** SCFM2, biofilm, AMR, *Pseudomonas aeruginosa*, evolution

## Abstract

Antimicrobial resistance (AMR) is a growing public health concern, particularly in biofilm-related infections, where microbial aggregates display high levels of tolerance. Oxidative stress has been hypothesized to accelerate the development of resistance, whereas antioxidants (AOs) may mitigate this process. In this study, we investigated the impact of AOs on the evolution of ciprofloxacin (CIP) resistance in *Pseudomonas aeruginosa* PAO1 using synthetic cystic fibrosis sputum medium (SCFM2), which mimics the physiochemical conditions of cystic fibrosis (CF) respiratory infections. Experimental evolution was performed over six passages with CIP alone or in combination with edaravone (ED), N-acetyl-cysteine (NAC), or thiourea (THU). Population analysis profiles and minimum inhibitory concentration (MIC) assays demonstrated that CIP treatment produced high-level resistance (MIC 8–32 mg/L), whereas CIP + AO treatments markedly suppressed resistance development (MIC 0.75–2 mg/L). Whole-genome sequencing revealed distinct mutational patterns. CIP-treated isolates carried mutations in *mexR* and *nalC* (efflux pump regulators), and *gyrA* (fluoroquinolone target), consistent with elevated resistance, along with additional mutations in *rocR* and *dnaX*. In contrast, evolved isolates in the presence of CIP + AO harbored *nfxB* mutations associated with lower resistance, while CIP + ED uniquely produced a mutation in *parS*. These findings support the role of reactive oxygen species (ROS) in driving resistance evolution under CF-like conditions and suggest that antioxidants can suppress this process, providing a potential strategy for limiting antimicrobial resistance in biofilm-associated infections.

**IMPORTANCE:**

Fighting antimicrobial resistance (AMR) is one of the greatest health challenges of our time. To find new ways to stop it, we need to better understand how resistance develops. Our study suggests that antioxidants may help slow down the process that allows bacteria to become resistant. We also show that resistance develops more quickly, and in a different way, when bacteria grow in conditions that resemble the human body rather than in standard laboratory media. In particular, the synthetic sputum medium promoted the formation of aggregated biofilms—sticky communities of cells that often occur in chronic and hard-to-treat infections.

## INTRODUCTION

Experimental evolution, which involves repeated cycles of bacterial growth, antimicrobial treatment, and environmental transfer, has become a powerful approach for studying the development of antimicrobial resistance (AMR) in microbial populations (1, 2). Within biofilms—structured microbial communities—bacteria occupy diverse ecological niches, leading to distinct, biofilm-specific pathways of AMR evolution and generating highly heterogeneous resistant populations (2, 3). Despite this, many chronic biofilm infections are not associated with surface-attached communities, but rather with bacterial aggregates embedded in host-derived substances such as mucus or tissue (3). Nevertheless, most *in vitro* models still rely on surface-attached biofilms, which fail to accurately replicate the complex biological and structural conditions of biofilm infections *in vivo* (2). The choice of a biofilm model has been shown to profoundly affect antimicrobial susceptibility outcomes, emphasizing the need for more physiologically relevant systems (2, 4).

To better replicate the microenvironment of cystic fibrosis (CF) lung infections—characterized by the presence of thick, viscous mucus in the airways—it is important to consider the complex composition of this mucus (5). Rich in mucins, DNA, and various nutrients such as carbohydrates, lipids, and amino acids, it provides an ideal habitat for the growth of pathogens like *Pseudomonas aeruginosa*, which is frequently isolated from the respiratory tracts of CF patients (6). *P. aeruginosa* biofilms show distinct gene expression *in vitro* and *in vivo*, affecting metabolism and increasing antibiotic tolerance (7). Studies show that genetically diverse *P. aeruginosa* populations exhibit a similar gene expression profile when grown under pathophysiological conditions, such as in CF sputum or explanted CF lung tissue (8, 9). Therefore, developing a synthetic medium that accurately mimics the biochemical composition and conditions of CF mucus has become essential for studying these infections under physiologically relevant conditions.

One example is the synthetic cystic fibrosis sputum medium (SCFM2), which closely replicates the nutrient composition of CF sputum and promotes biofilm aggregate formation (10). *P. aeruginosa* grown in SCFM2 exhibits gene expression and physiological traits closely resembling those seen in CF patient sputum, making it a valuable model for studying AMR in a clinically relevant setting (11, 12). It is enriched with components, such as salmon sperm DNA, dioleoylphosphatidylcholine (DOPC), *N*-acetylglucosamine, and mucin, which enhance viscosity and facilitate the formation of biofilm aggregates embedded within the medium as in CF lungs (11, 13). The utility of SCFM2 in experimental evolution has been exemplified by studies investigating the use of the quorum-sensing inhibitor (QSI) furanone C-30 in combination with tobramycin. Although initially effective, resistance to this combination emerged rapidly, with whole-genome sequencing (WGS) revealing mutations in *mexT*, *fusA1*, and *parS*—genes associated with antibiotic resistance. Notably, similar mutations were also found in populations treated with QSI alone, suggesting a limited evolutionary barrier to resistance even in QSI-based therapies (14). Complementary *in vivo* models have provided additional insights into evolution of AMR under biofilm-associated growth conditions. A murine model of biofilm lung infection, in which bacteria were embedded in alginate beads and exposed to sub-inhibitory concentrations of CIP, revealed a rapid onset of resistance driven primarily by efflux pump mutations. Inflammation was also elevated during early infection stages, implicating host-derived reactive oxygen species (ROS) as potential contributors to mutagenesis and AMR development (15).

Sub-inhibitory bactericidal antibiotic concentrations, such as those of CIP, can enhance ROS production through mechanisms involving DNA gyrase inhibition and the Fenton reaction, ultimately increasing oxidative stress and mutation rates (15, 16). Given the link between oxidative stress and AMR evolution (17, 18), we explored the use of antioxidants (AOs) as potential modulators of resistance development. In a previous study, we investigated the effects of combining CIP with AOs, including N-acetylcysteine (NAC), edaravone (ED), and thiourea (THU), in two *in vitro* models: surface-attached *P. aeruginosa* biofilms grown in flow cells and static biofilms on glass beads (17). While the CIP + AO combinations reduced resistance emergence in both models, the effect was more pronounced in flow-cell biofilms. Among the AOs, ED and THU outperformed NAC in suppressing ROS accumulation and resistance development. These findings underscore the importance of both the biofilm model and AO selection when evaluating AMR mitigation strategies. To further explore these observations under more physiologically relevant conditions, we employed the SCFM2 medium, which closely mimics the nutritional and chemical environment of CF lung, to assess whether AOs could similarly attenuate the evolution of AMR in this context.

**Fig 1:**
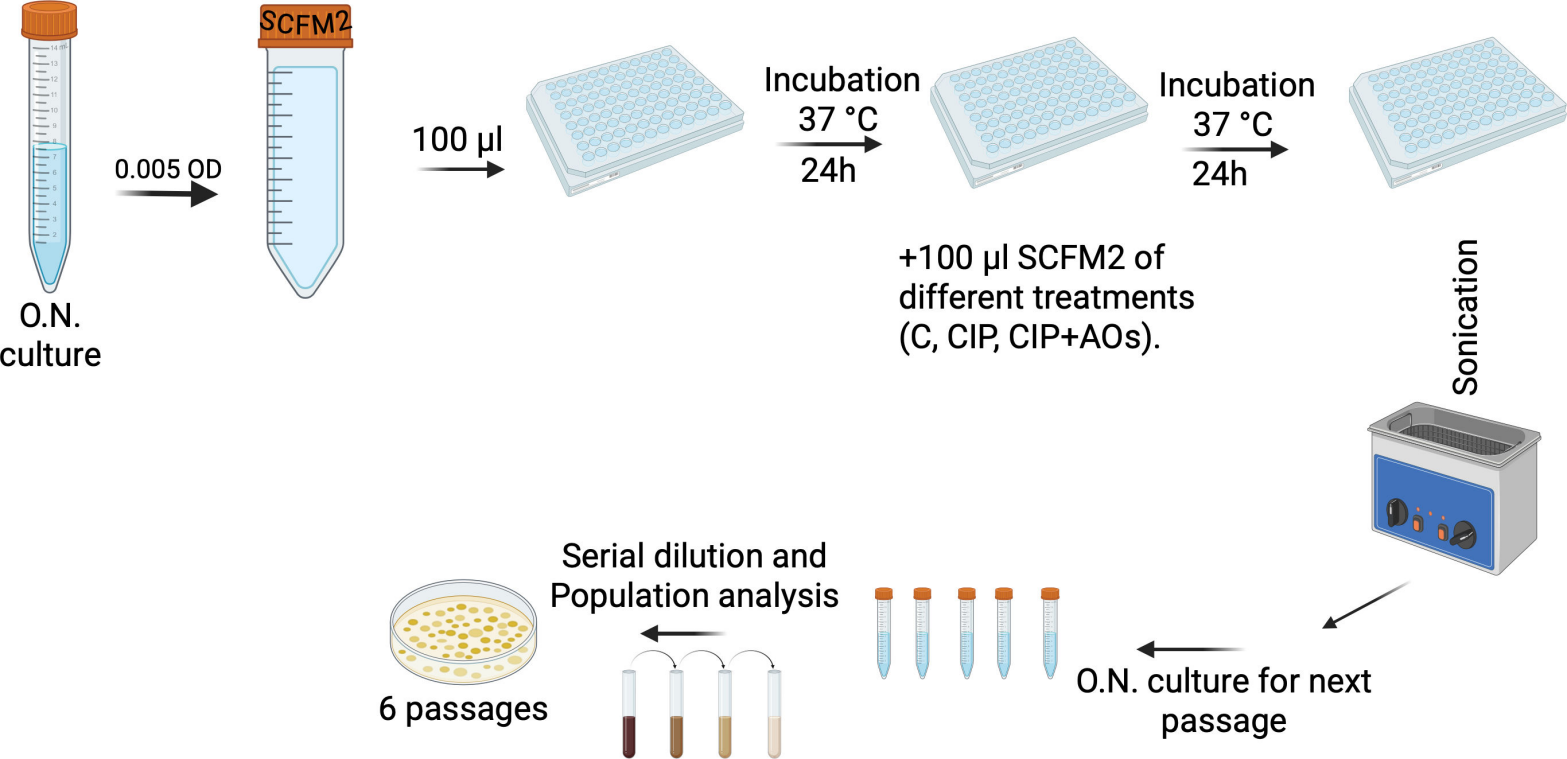
Schematic illustration of the evolution study. An overnight culture of *P. aeruginosa* PAO1 was grown at 37°C in LB medium and diluted to inoculate SCFM2. In a 96-well plate, 100 µL of the inoculated medium was added to each well, followed by incubation at 37°C overnight. The experimental different conditions included SCFM2 treatment with ciprofloxacin alone or in combination with antioxidants (CIP + AOs), including ED, NAC, or THU. The evolution experiment was conducted over six continuous passages.

In this study, we performed experimental evolution of *P. aeruginosa* PAO1 in SCFM2 during treatment with either CIP alone or in combination with NAC, ED, or THU for 6 passages (Fig. 1). Resistance development was tracked through population analysis, minimum inhibitory concentration (MIC) testing, and WGS. Our findings confirm the capacity of AOs to reduce the evolution of CIP resistance in SCFM2-grown biofilms, although the effectiveness varied by the type of AO. Additionally, resistant isolates exhibited high levels of MICs, and the mutations observed differed from those in surface-attached models. Overall, our results highlight the utility of SCFM2 as a physiologically relevant model for studying AMR and demonstrate the potential of antioxidant co-therapies to mitigate resistance development in *P. aeruginosa* biofilms.

## MATERIALS AND METHODS

### Bacterial strains, antibiotics, antioxidants, and culture media

The overnight culture of *P. aeruginosa* wild-type PAO1 was initiated from a frozen stock at −80°C in LB medium under aerobic conditions at 37°C. The SCFM2 medium was prepared as previously detailed (13). Briefly, SCFM2 was prepared by supplementing SCFM (11) with macromolecules commonly found in CF sputum. Specifically, salmon sperm DNA (600 µg mL⁻¹), N-acetylglucosamine (300 µM), bovine maxillary mucin (5 mg mL⁻¹), and the lipid DOPC (100 µg mL⁻¹) were added. The resulting SCFM2 thus mimicked the biochemical and macromolecular composition of CF sputum. To assess the ability of AOs to inactivate ROS and slow down the development of CIP resistance, three AOs were tested: edaravone (ED; 3-methyl-1-phenyl-2-pyrazolin-5-one) (Merck, Denmark), N-acetylcysteine (NAC) (Sigma, USA), and thiourea (THU) (Sigma, USA). The AOs were used at the following concentrations: ED 300 mM, NAC 2 mM, and THU 100 mM, which have previously been tested for their ability to reduce ROS levels (17). For experimental evolution, ciprofloxacin (CIP) (a fluoroquinolone antibiotic) was used at 0.25 mg/L in SCFM2. The same CIP concentration was used across all experimental conditions, including those with antioxidant combinations.

### Evolution experiment

Overnight cultures of *P. aeruginosa* PAO1 grown at 37°C in LB were diluted to a final inoculum of 5 × 10⁷ CFU/mL in SCFM2 medium. Then, 100 µL of the inoculum was dispensed into a U-bottom 96-well plate (Greiner Bio-One, Frickenhausen, Germany) and incubated overnight at 37°C without shaking to allow aggregate formation. After 24 h, 100 µL of fresh SCFM2 medium containing CIP or CIP combined with one of the three AOs (ED, NAC, or THU) was prepared in SCFM2 at the specified concentrations and was carefully added to the edge of each well, bringing the total volume to 200 μL. Control wells received fresh, CIP-free SCFM2 medium. Six independent lineages represented each condition. The plate was then incubated overnight at 37°C, followed by sonication for 5 min and vortexing at 900 rpm for another 5 min. Then, 30 μL of the bacterial suspension was used to initiate an overnight culture to start a new passage, while the remaining volume was stored at −80°C in glycerol. This was repeated for six continuous passages. A scheme describing the workflow of the evolution study is shown in Fig. 1.

### Determination of minimum inhibitory concentrations

The MIC of CIP was determined using the broth microdilution method. A stock solution of CIP (2 mg/mL) was diluted to 64 mg/L in Mueller-Hinton broth medium (MH). A two-fold serial dilution was performed in a flat 96-well microtiter plate. Overnight bacterial cultures of the evolved lineages of different bacterial populations were prepared and incubated at 37°C with shaking. The optical density (OD) at 600 nm was measured and adjusted to an OD corresponding to 1 × 10^8^ CFU/mL in MH medium. This suspension was diluted 1:100 in MH medium. Each well in columns 1–10 received 100 µL of the bacterial inoculum to achieve a final CIP concentration range of 32–0.06 mg/L in a total volume of 200 μL per well. Column 11 received 200 µL of CIP-free bacterial inoculum, while column 12 received 200 µL of MH medium as a blank control. Plates were incubated at 37°C for 24 h, and then, the absorbance was measured at 600 nm with a microplate reader. And then the MIC of CIP that inhibits the growth of 50% of the bacterial populations (MIC_50_) was calculated. The MIC of single bacterial colonies (isolates) selected from the different evolved populations was determined using E-test strips (bioMérieux SA, France). The biofilm prevention concentration (BPC) is defined as the lowest concentration of CIP that prevents the formation of a detectable biofilm, typically reflected by no measurable increase in biofilm biomass or viable cells compared with the baseline (inoculum-only) control. The minimum biofilm inhibitory concentration (MBIC) was calculated as the lowest CIP concentration that produced a ≥2-log₁₀ (≥99%) reduction in viable biofilm-associated *P. aeruginosa* cells compared with the untreated biofilm control.

### Population analysis profiles (PAP)

Overnight cultures were prepared from the evolved bacterial populations subjected to different treatments across the six different passages (six lineages per condition), i.e., a total of 30 populations per evolution passage. These cultures were serially diluted and plated on LB agar plates supplemented with varying concentrations of CIP (0, 0.1, 0.2, 0.5, and 1 mg/L). The proportion of the resistant subpopulation was determined by dividing the number of colony-forming units (CFU) on CIP-supplemented plates by the total CFU of the population grown on CIP-free LB plates.

### Growth rates of CIP-resistant isolates

At the sixth (final) passage of the evolution experiment, four bacterial isolates were collected from plates with bacterial growth on the highest CIP concentration for each treatment condition. Overnight cultures of these isolates were prepared in LB medium, standardized to an OD_600_ of 0.1, and subsequently diluted 1,000-fold. Next, 100 μL of each diluted culture was added in triplicate to individual wells of a 96-well flat-bottomed plate (Nunc, Thermo Fisher Scientific). The plate was incubated at 37°C with shaking at 225 rpm for 24 h in an Infinite F200 Pro plate reader (Tecan), with the lid on. Absorbance (OD_600_ nm) was measured every 20 min throughout the 24-hour incubation using Magellan V 7.2 software. Growth rates were determined from the resulting growth curves by applying the Gompertz fitting model.

### Microscopy

The PAO1 strain was either cultured in SCFM2 medium or in LB medium in a 96-well imaging plate (zell-kontakt GmbH, Germany) and incubated overnight at 37°C. The wells were stained with Syto9 (ThermoFisher, USA), and images were captured using the confocal microscopy Zeiss 880 (Plan-Apochromat 63/1.40 oil differential interference contrast [DIC] objective; for GFP fluorescence, excitation at 488 nm and emission range 493–587). A minimum of three z-stacks was captured for the green channel. Minimal z-stack image processing (smoothing, background subtraction, and different three-dimensional [3D] views) and the 3D photo statistics were performed using Imaris 10.1 software (Bitplane AG, Zurich, Switzerland).

### Whole-genome sequencing

In the final passage (passage 6) of the SCFM2 evolution experiment, four isolates and two lineage populations per condition (a total of 20 isolates and 10 evolved populations) were selected for WGS. DNA was extracted from an overnight culture using a DNeasy Blood & Tissue kit (Qiagen, Netherlands), according to the manufacturer’s instructions. Libraries were prepared following the Hackflex workflow (19) and were sequenced on a NovaSeq 6000 instrument. The sequenced reads were assessed for quality using FastQC and aligned to the *P. aeruginosa* PAO1 reference genome (GenBank accession: AE004091) using Bowtie2. Variants were called with BCFtools and filtered based on a quality score (QUAL) >10, a read depth (DP) >10, and an allele frequency >20%. Finally, the variants were annotated using SnpEff.

### Statistics

The population analysis was conducted using two-way ANOVA followed by Tukey’s multiple comparison test. Unpaired *t*-tests were used in the analysis (Prism 10, GraphPad Software, San Diego, USA).

## RESULTS

### Evolution of AMR and biofilm formation

Building on our previous investigation of the effect of AOs on the development of antibiotic resistance (17), the wild-type *P. aeruginosa* PAO1 strain was experimentally evolved in six consecutive passages. In each passage, *P. aeruginosa* PAO1 was exposed to CIP (0.25 mg/L), which corresponds to 1/4 of the BPC and 1/8 of the MBIC, or to CIP combined with AOs (CIP + AOs) in SCFM2 medium, which resembles CF sputum in patients (Fig. 1). Combining CIP with different AOs did not alter its activity against PAO1. The MIC remained identical to that of CIP alone (0.125 mg/L).

**Fig 2:**
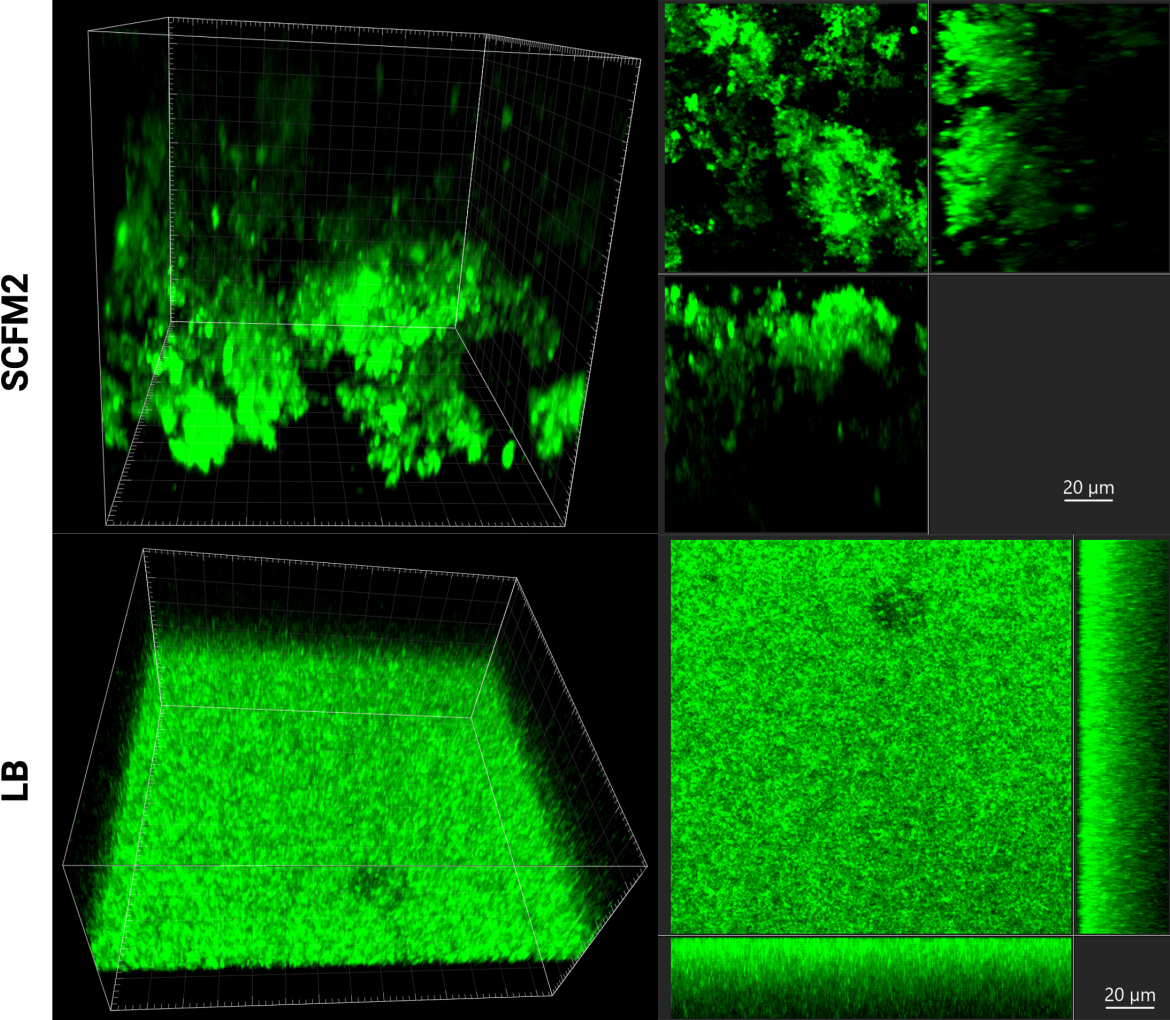
Growth of *P. aeruginosa* PAO1 in SCFM2 (upper row) and LB medium (lower row) as visualized with microscopy after staining with Syto9. Z-stacks 3D (left panel) and sections (right panel) are shown for each condition.

To assess microbial aggregation in SCFM2 compared to LB medium, cells were grown overnight in a microtiter plate following the same growth conditions, then stained with Syto9, which fluoresces nucleic acids in green and allows cell morphology visualization. Imaging revealed different modes of growth between the two media, with cells growing in the SCFM2 medium forming structured aggregates, while those grown in LB medium displayed a more flatter arrangement (Fig. 2).

### Antioxidants diminish antimicrobial resistance to CIP in SCFM2

**Fig 3:**
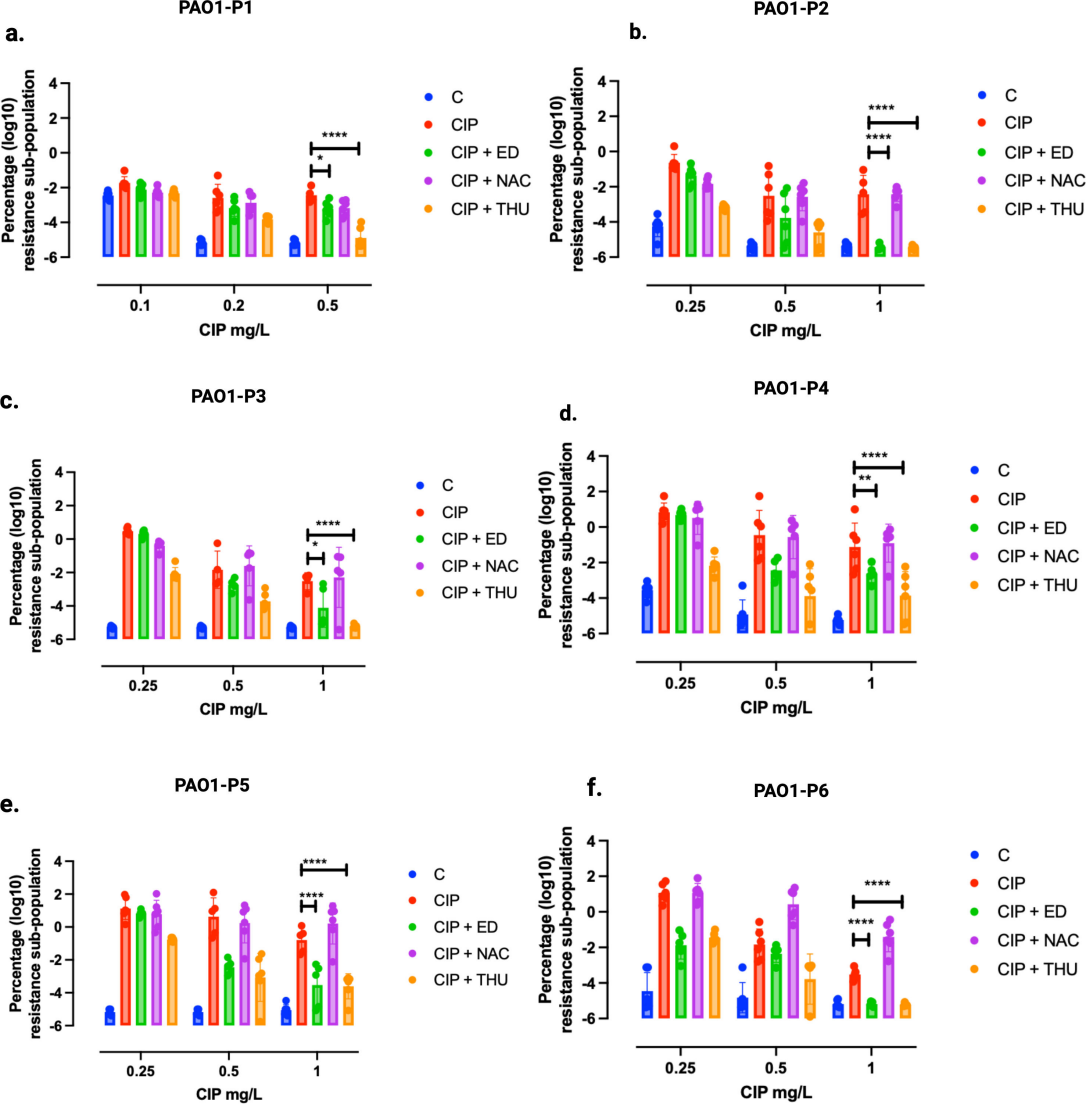
Population analysis of the SCFM2 evolution experiment. Harvested evolved populations from the different treatment conditions of SCFM2 in 96-well plates were used to start an overnight culture. The biofilm populations in the six passages were treated with 0.25 mg/L CIP, CIP + ED, CIP + NAC, or CIP + THU, with untreated biofilms as a control (C). The percentage of surviving bacteria at different CIP concentrations (0.1, 0.2, 0.5, and 1 mg/L) is shown as log10 values. (**a–f**) Passages P1 to P6. The levels of statistical significance are represented as follows: *P* < 0.05 (*), *P* < 0.01 (**), and *P* < 0.0001 (****).

The harvested populations from each passage were analyzed using PAP to assess resistance development by plating them on medium supplemented with CIP at varying concentrations (0.1, 0.2, 0.5, and 1 mg/L) (Fig. 3a through f). PAP analysis revealed significant differences in the surviving sub-population among the treatment conditions C, CIP + ED, and CIP + THU compared to CIP alone across different evolution passages in the SCFM2 medium. Surprisingly, the combination of CIP + NAC did not contribute to a reduction in the development of AMR relative to CIP. Surviving subpopulations at 0.5 mg/L CIP in the first passage revealed significant reduction in those evolved under CIP + ED (*P* = 0.0327) or CIP + THU (*P* < 0.0001) compared to CIP alone (Fig. 3). This trend persisted through subsequent passages, with varying levels of significance, reaching *P* < 0.0001 for most comparisons at a CIP concentration of 1 mg/L.

**Fig 4:**
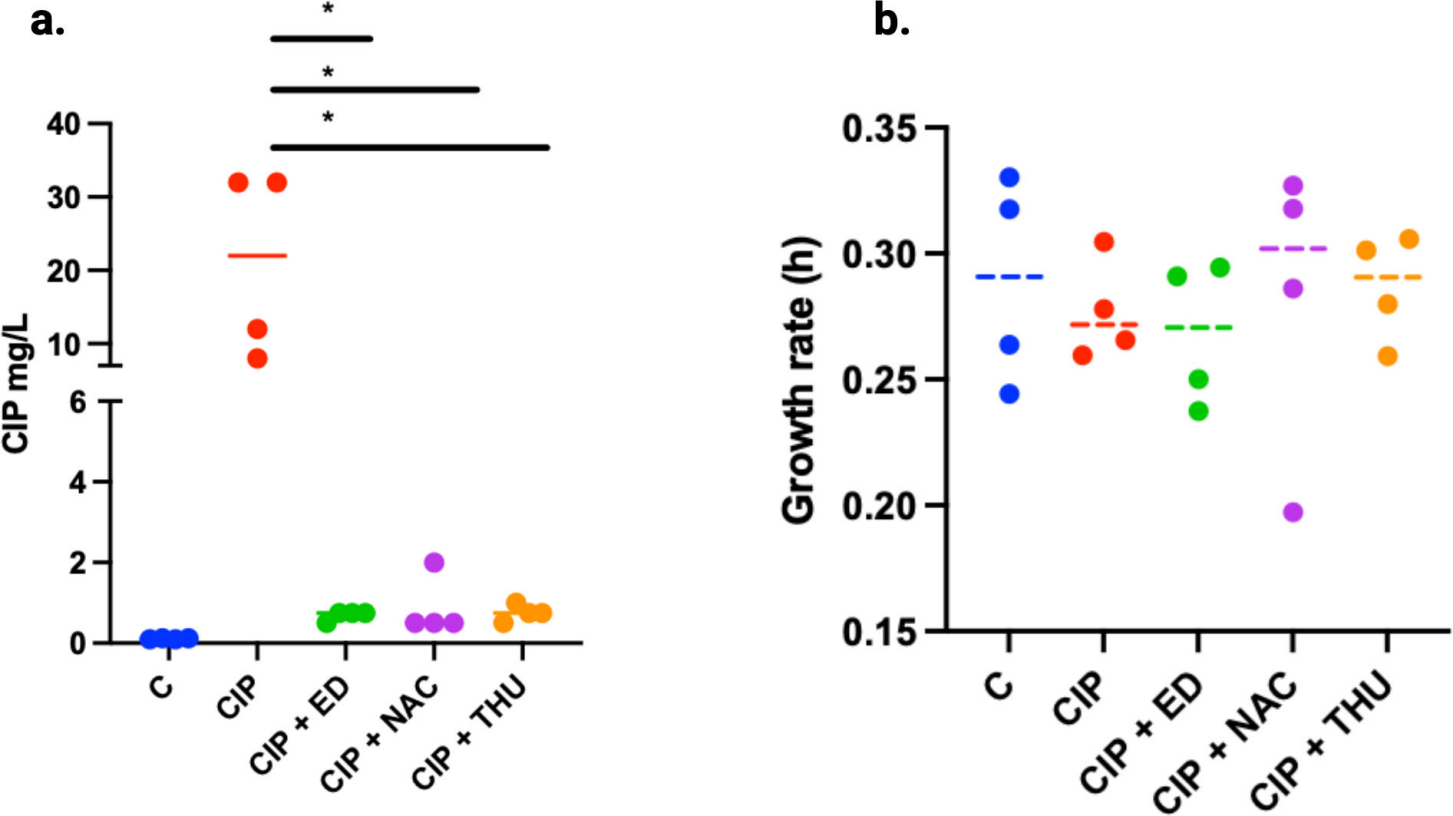
Effect of AO treatments on CIP MICs and growth rates of PAO1. (**a**) Four isolates per condition were selected from the population analysis (6^th^ passage), and the MIC levels were determined using E-test (*P < 0.05). (**b**) The same isolates were tested for their growth rate in LB, calculated from growth curves. Control in (blue), CIP (red), CIP + ED (green), CIP + NAC (purple), and CIP + THU (orange). The levels of statistical significance, determined using a *t*-test, are represented as follows: *P* < 0.05 (*).

The MIC of CIP was determined against the different evolved populations of the sixth passage, and the MIC50 was calculated (Table S1). The control population C revealed an MIC_50_ of 0.125 mg/L. In contrast, CIP-treated populations displayed an increased MIC_50_ of 1 mg/L. The addition of antioxidant ED reduced the MIC_50_ to 0.25 mg/L, while the combination of CIP with either THU or NAC resulted in populations with an MIC_50_ of 0.5 mg/L. Subsequently, CIP MICs were determined using E-test for isolates selected from the surviving subpopulations of the different treatment conditions in passage 6 of the PAP assay (Fig. 4a). The isolates from the control population exhibited low MIC values (0.1 mg/L). In contrast, the isolates evolved under CIP treatment exhibited significantly higher MICs (8–32 mg/L) compared to those evolved under CIP + AOs (*P* < 0.05). MICs for isolates from evolved populations exposed to CIP + NAC ranged from (0.5 to 2 mg/L), CIP + ED from (0.5 to 0.75 mg/L), and CIP + THU from (0.75 to 1 mg/L). This demonstrates substantial resistance development in the CIP-treated bacterial populations, which was diminished by the addition of AOs. No significant differences in growth rates of the CIP-resistant isolates were observed (Fig. 4b).

### Mutations occurring during evolution under different treatment conditions

**Fig 5:**
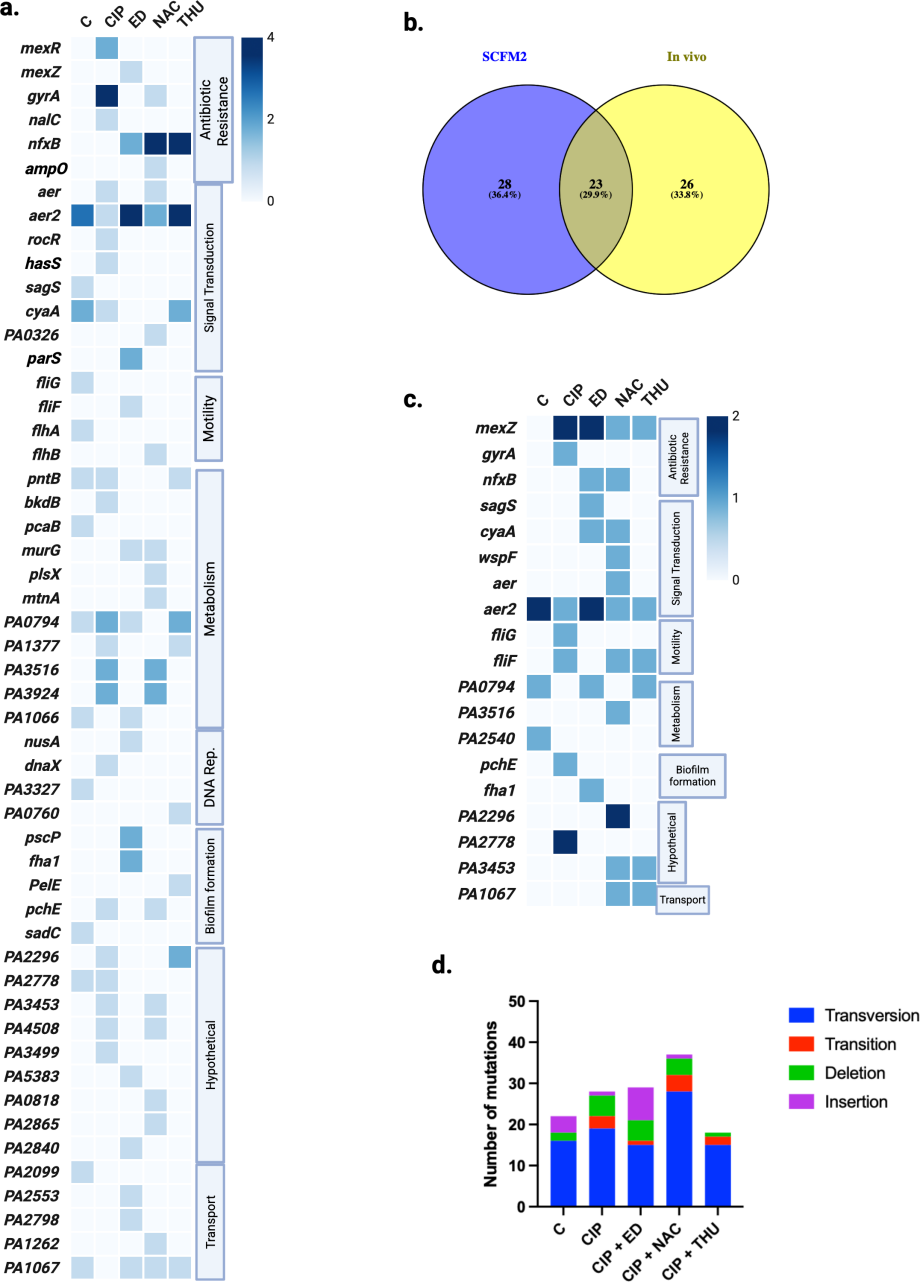
Mutation analysis of different treatment conditions. (**a**) Whole-genome sequencing was performed for four selected isolates from each treatment condition (sixth passage) and presented in a heatmap. The mutated genes are categorized by function, including antibiotic resistance, signal transduction, motility, metabolism, DNA replication, biofilm formation, hypothetical proteins, and transport. The intensity of the blue gradient represents the number of isolates exhibiting the same mutated genes. Conditions: Control (C), ciprofloxacin (CIP), ciprofloxacin combined with edaravone (CIP + ED), ciprofloxacin combined with N-acetylcysteine (CIP + NAC), ciprofloxacin combined with thiourea (CIP + THU) (File S1 and File S2) (**b**) Venn diagram showing the overlap of mutations in SCFM2 evolution experiments (isolates) vs. *in vivo* (15) evolution experiment isolates (Table S2 and File S2 ). (**c**) Heatmap of mutations for the whole-genome sequenced populations (sixth passage) under different treatment conditions (File S3). (**d**) Bar graph of mutation types observed under different conditions (populations and selected isolates). Mutation types are categorized as transversions (blue), transitions (red), deletions (green), and insertions (purple). The WGS analysis output Excel sheet can be found in (File S4).

The WGS was performed for four isolates and two populations per treatment condition, with the populations included to identify mutations that were most persistent or occurred at high frequencies within the population. The results indicate that the evolved populations and selected isolates during CIP treatment exhibited the highest number of distinct mutated genes (18 vs. 9 in C; 20 vs. 12 in CIP + ED; and 15 vs. 8 in CIP + THU) (Fig. S1). In contrast, the CIP + NAC treatment did not show a similar pattern. We present an overview of the observed mutations in selected isolates in (Fig. 5a). Mutations were observed in genes, including *mexR*, *mexZ*, *nalC*, *ampO*, *nfxB*, and *gyrA*. Mutations in *nalC* and *mexR* were only observed in CIP-treated isolates. A *gyrA* transition mutation (248 C>T), resulting in an amino acid change from threonine to isoleucine at position 83 (Thr83Ile), was observed in all four isolates after CIP treatment. A deletion in *rocR* was found in isolates that evolved during CIP treatment, but was absent in those evolved during CIP + AO treatments. Genes such as *fliG*, *fliF*, *flhA*, and *flhB* exhibited treatment-specific mutational changes with a broader distribution across the experimental conditions. The *pscP* and *fha1* genes were specifically mutated in isolates exposed to CIP + ED combined treatment. The mutations observed in the isolates from the SCFM2 evolution study were subsequently compared with mutations that emerged during *in vivo* mouse lung infections (15) to assess the ability of the SCFM2 medium to replicate *in vivo* conditions (Fig. 5b). The analysis revealed that mutations in 23 common genes were exhibited in both studies (Table S2). Among the shared mutated genes, those that appeared exclusively during CIP treatment—both *in vivo* (CIP treatment) and in SCFM2 (either with CIP alone or in combination with AOs)—suggest a selective pressure imposed by CIP, driving specific adaptive changes. For example, mutations in *nfxB*, which regulates the MexCD-OprJ efflux pump, were observed in evolved isolates from both studies, along with mutations in the sensory histidine kinase gene *parS* and the iron siderophore sensor protein *hasS* gene. Additionally, two genes associated with biofilm formation, *pchE* and *pscP,* exhibited mutations in both SCFM2 and *in vivo* conditions.

A heatmap of the mutations identified in evolved biofilm populations from the sixth passage—where the isolates were selected—is presented in (Fig. 5c). A transition mutation (248 C>T) in the *gyrA* gene was detected in the CIP-treated populations with an allele frequency of 32% in the sequenced population. Similarly, a transversion mutation (3887 A>C) in the *pchE* gene, resulting in an amino acid change from glutamine to proline at position 1296 (Gln1296Pro), exhibited an allele frequency of 42% during CIP treatment. In addition, a deletion mutation in *fliG* and a transition mutation in *fliF* showed allele frequencies of 20% and 18%, respectively. The distribution of mutation types across all conditions, for both evolved populations and selected isolates, is shown in (Fig. 5d). In every condition, transversion mutations occurred more frequently than transitions, comprising 72% in C, 67% in CIP, 51% in ED, 75% in NAC, and 83% in THU.

## DISCUSSION

The fast development of CIP resistance in SCFM2—a medium that promotes *P. aeruginosa* aggregation—is consistent with previous studies showing that CIP resistance in PAO1 arises more rapidly in a colony biofilm model than in LB stationary cultures (1). SCFM2 medium was selected for its ability to better mimic the *in vivo* environment, particularly by promoting biofilm growth, which is critical in the context of chronic infections in clinical settings.

In the current study, CIP-resistant isolates with high MICs (32 µg/mL) emerged during evolution in SCFM2 under CIP exposure. This was previously observed in PAO1 Δ*katA* but not in wild-type PAO1 after experimental evolution conducted in the presence of CIP in the colony biofilm model (1). As Δ*katA* mutants experience higher oxidative stress in the presence of CIP than the wild-type PAO1, we might speculate that the bacterial aggregates formed in SCFM2 are exposed to higher oxidative stress than cells in the colony biofilm model. This confirms the specificity of the evolutionary pathways toward AMR in the different biofilm models. The high CIP MICs were associated with mutations in the target gene *gyrA*, along with mutations in *nalC* and *mexR* (repressor of the MexAB-oprM efflux pump), in CIP-evolved isolates. A *gyrA* (encoding gyrase subunit) transition mutation, which plays a role in reducing fluoroquinolone binding (20), was observed in all four isolates after CIP treatment. A deletion in *rocR,* which encodes a c-di-GMP phosphodiesterase involved in biofilm formation (21), was identified in isolates evolved during CIP treatment but was absent in those from CIP + AO treatment. Genes associated with motility functions, such as *fliG*, *fliF*, *flhA*, and *flhB,* exhibit treatment-specific mutational changes with a broader distribution across the experimental conditions. The *pscP gene,* encoding a structural component of the type III secretion system (22), and the *fha1* gene, playing a major role in biofilm formation (23), were specifically mutated in isolates exposed to CIP + ED combined treatment.

The CIP-resistant isolates selected from populations evolved in the presence of CIP + AO presented mutations only in *nfxB* and *mexZ* regulators of efflux pumps, which are associated with lower MIC levels compared to isolates evolved during CIP alone. Interestingly, mutations in these two genes were also found in *P. aeruginosa* isolates with low-level resistance to CIP selected during the *in vivo* experimental evolution in the animal model of biofilm lung infection (15). Investigation of the growth rates of CIP-resistant isolates showed no growth defects associated with these mutations, probably due to compensatory mutations, as was shown in a previous study (24). The present PAP results show a slower development of resistance to CIP in populations exposed to the combinations of CIP + THU and CIP + ED than to CIP + NAC, which did not decrease the development of AMR. This is in line with previously published results showing that NAC caused a moderate decrease in ROS formation during CIP treatment compared to ED and THU, probably due to the different mechanisms of action of these AOs (17). However, NAC was shown to reduce the occurrence of mutations in *nfxB*, which cause CIP resistance in the flow-cell model system (17), underlining the different evolutionary outcomes observed in different biofilm models. These findings highlight the crucial role of the growth environment in influencing resistance development and emphasize the variability of adaptive responses based on the growth medium used.

The main reason for conducting the experimental evolution in SCFM2 was to try to mimic the *in vivo* conditions. To this end, we analyzed nonsynonymous gene mutations that emerged in the final passage (6th passage) of our SCFM2 evolution experiment and compared them to mutations identified in a previous *in vivo* biofilm evolution study, in which alginate-embedded *P. aeruginosa* evolved over four passages in mouse lungs (4th passage). Although both models are obviously different, we observed an overlap in mutated metabolic genes between the SCFM2 evolution experiment and the *in vivo* study. The presence of 23 commonly mutated genes in both SCFM2-evolved isolates and *in vivo* mouse lung infections indicates a strong degree of evolutionary convergence under CIP pressure. This overlap suggests that SCFM2 medium can replicate key environmental and selective conditions found in the host, supporting its utility as a model for biofilm infections. Several of these mutations—particularly in *nfxB, parS*, and *hasS*—were exclusive to CIP-treated conditions in both systems, highlighting the consistent selective pressure exerted by CIP across environments. In addition to resistance-associated genes, shared mutations in *pchE* and *pscP*, which are involved in biofilm formation, suggest that adaptive changes to biofilm structure or regulation are a common feature of CIP-driven evolution. Together, these findings reinforce the relevance of SCFM2 as an experimental platform for studying resistance evolution and the genetic basis of persistence in biofilm-associated infections. When bacteria are exposed to bactericidal antibiotics such as β-lactams, aminoglycosides, and quinolones, drug–target interactions stimulate increased NADH oxidation through the tricarboxylic acid (TCA) cycle and the electron transport chain (25). This heightened metabolic activity leads to excessive production of ROS, including superoxide and hydroxyl radicals (•OH). The hydroxyl radicals, formed primarily via the Fenton reaction, cause extensive damage to DNA, proteins, lipids, and nucleotide pools, ultimately contributing to cell death. In response to ROS-induced DNA damage, bacteria activate the SOS response—an error-prone DNA repair pathway that can introduce mutations. The SOS was found in different studies to be triggered by the use of sub-MIC of different antibiotics (26). These mutations may accumulate over time, driving the evolution of antibiotic resistance through mechanisms, such as target modification, enhanced efflux pump expression, and decreased drug uptake (25). Exposure of uropathogenic *Escherichia coli* (UPEC) to sub-inhibitory concentrations of SOS-inducing antibiotics can activate the bacterial SOS response, leading to increased expression of integrase genes encoded within pathogenicity islands (PAIs). These phage-like integrases mediate excision events, resulting in the loss or rearrangement of PAIs and contributing to genome plasticity (27). This finding correlates with the broader concept that antibiotic-induced oxidative stress and DNA damage stimulate the SOS response, an error-prone repair system that promotes both mutagenesis and genomic rearrangements. Consequently, while such antibiotics are designed to eliminate pathogens, their sub-lethal exposure may inadvertently enhance bacterial adaptability by facilitating genetic diversification and potentially influencing antibiotic resistance. The use of AOs has been proposed as a potential strategy to mitigate ROS-mediated DNA damage, thereby reducing mutation rates and possibly slowing the development of antibiotic resistance (28). Comparing the evolutionary trajectories that occur in different biofilm models under selection with CIP or CIP + AO, we can conclude that CIP resistance was selected both in flow-cell, glass beads (17), and in SCFM2 biofilm models, and that AO had a mitigating effect, as shown by population MICs and PAP analysis. Based on the present results, an *in vivo* experimental evolution model might be utilized to test the effect of AOs such as ED combined with CIP treatment in reducing resistance development. In summary, our findings demonstrate that the development of CIP resistance in *P. aeruginosa* is strongly influenced by the growth environment, with SCFM2—an *in vitro* model mimicking the *in vivo* biofilm conditions—promoting fast and more clinically relevant resistance evolution. The emergence of high-level resistance in SCFM2 was linked to mutations in key resistance-associated genes, such as *gyrA*, *mexR*, and *nalC*, and showed significant overlap with mutations observed in an *in vivo* lung infection model, indicating evolutionary convergence. Additionally, antioxidant combinations such as CIP + ED or CIP + THU slowed resistance development, while NAC had a limited effect. These results highlight the importance of using physiologically relevant biofilm models for studying AMR and support the potential of combining antibiotics with adjuvants, such as ED, to mitigate resistance in clinical settings.

## Data Availability

All the data for the sequencing results can be accessed through the SRA project under the accession number PRJNA1306673.

## SUPPLEMENTAL MATERIAL

Data S1 spectrum.03149-25-s0001.xlsx Mutated gene isolates passage 6.

Data S2 spectrum.03149-25-s0002.xlsx Number of isolates with mutated genes; list of common and unique mutated genes.

Data S3 spectrum.03149-25-s0003.xlsx Mutated genes in populations passage 6.

Data S4 spectrum.03149-25-s0004.xlsx WGS analysis.

Supplemental material spectrum.03149-25-s0005.docx Tables S1 and S2; Fig. S1.

ASM does not own the copyrights to Supplemental Material that may be linked to, or accessed through, an article. The authors have granted ASM a non-exclusive, world-wide license to publish the Supplemental Material files. Please contact the corresponding author directly for reuse.

## References

[1] Ahmed MN, Porse A, Sommer MOA, Høiby N, Ciofu O. 2018. Evolution of antibiotic resistance in biofilm and planktonic Pseudomonas aeruginosa populations exposed to subinhibitory levels of ciprofloxacin. Antimicrob Agents Chemother 62:e00320-18. doi:10.1128/AAC.00320-18 29760140 PMC6105853

[2] Coenye T, Bové M, Bjarnsholt T. 2022. Biofilm antimicrobial susceptibility through an experimental evolutionary lens. NPJ Biofilms Microbiomes 8:82. doi:10.1038/s41522-022-00346-4 36257971 PMC9579162

[3] Ciofu O, Moser C, Jensen PØ, Høiby N. 2022. Tolerance and resistance of microbial biofilms. Nat Rev Microbiol 20:621–635. doi:10.1038/s41579-022-00682-4 35115704

[4] Rumbaugh KP, Whiteley M. 2025. Towards improved biofilm models. Nat Rev Microbiol 23:57–66. doi:10.1038/s41579-024-01086-2 39112554

[5] Morrison CB, Markovetz MR, Ehre C. 2019. Mucus, mucins, and cystic fibrosis. Pediatr Pulmonol 54 Suppl 3:S84–S96. doi:10.1002/ppul.24530 31715083 PMC6853602

[6] Bhagirath AY, Li Y, Somayajula D, Dadashi M, Badr S, Duan K. 2016. Cystic fibrosis lung environment and Pseudomonas aeruginosa infection. BMC Pulm Med 16:174. doi:10.1186/s12890-016-0339-5 27919253 PMC5139081

[7] Cornforth DM, Dees JL, Ibberson CB, Huse HK, Mathiesen IH, Kirketerp-Møller K, Wolcott RD, Rumbaugh KP, Bjarnsholt T, Whiteley M. 2018. Pseudomonas aeruginosa transcriptome during human infection. Proc Natl Acad Sci USA 115:E5125–E5134. doi:10.1073/pnas.1717525115 29760087 PMC5984494

[8] Rossi E, Falcone M, Molin S, Johansen HK. 2018. High-resolution in situ transcriptomics of Pseudomonas aeruginosa unveils genotype independent patho-phenotypes in cystic fibrosis lungs. Nat Commun 9:3459. doi:10.1038/s41467-018-05944-5 30150613 PMC6110831

[9] Kordes A, Preusse M, Willger SD, Braubach P, Jonigk D, Haverich A, Warnecke G, Häussler S. 2019. Genetically diverse Pseudomonas aeruginosa populations display similar transcriptomic profiles in a cystic fibrosis explanted lung. Nat Commun 10:3397. doi:10.1038/s41467-019-11414-3 31363089 PMC6667473

[10] De Bleeckere A, Van den Bossche S, De Sutter P-J, Beirens T, Crabbé A, Coenye T. 2023. High throughput determination of the biofilm prevention concentration for Pseudomonas aeruginosa biofilms using a synthetic cystic fibrosis sputum medium. Biofilm 5:100106. doi:10.1016/j.bioflm.2023.100106 36845825 PMC9945637

[11] Palmer KL, Aye LM, Whiteley M. 2007. Nutritional cues control Pseudomonas aeruginosa multicellular behavior in cystic fibrosis sputum. J Bacteriol 189:8079–8087. doi:10.1128/JB.01138-07 17873029 PMC2168676

[12] Cornforth DM, Diggle FL, Melvin JA, Bomberger JM, Whiteley M. 2020. Quantitative framework for model evaluation in microbiology research using Pseudomonas aeruginosa and cystic fibrosis infection as a test case. mBio 11:e03042-19. doi:10.1128/mBio.03042-19 31937646 PMC6960289

[13] Turner KH, Wessel AK, Palmer GC, Murray JL, Whiteley M. 2015. Essential genome of Pseudomonas aeruginosa in cystic fibrosis sputum. Proc Natl Acad Sci USA 112:4110–4115. doi:10.1073/pnas.1419677112 25775563 PMC4386324

[14] Bové M, Bao X, Sass A, Crabbé A, Coenye T. 2021. The quorum-sensing inhibitor furanone C-30 Rapidly loses its tobramycin-potentiating activity against Pseudomonas aeruginosa biofilms during experimental evolution. Antimicrob Agents Chemother 65:e0041321. doi:10.1128/AAC.00413-21 33903100 PMC8373219

[15] Higazy D, Pham AD, van Hasselt C, Høiby N, Jelsbak L, Moser C, Ciofu O. 2024. In vivo evolution of antimicrobial resistance in a biofilm model of Pseudomonas aeruginosa lung infection. ISME J 18:wrae036. doi:10.1093/ismejo/wrae036 38478426 PMC10980832

[16] Kohanski MA, DePristo MA, Collins JJ. 2010. Sublethal antibiotic treatment leads to multidrug resistance via radical-induced mutagenesis. Mol Cell 37:311–320. doi:10.1016/j.molcel.2010.01.003 20159551 PMC2840266

[17] Higazy D, Ahmed MN, Ciofu O. 2024. The impact of antioxidant-ciprofloxacin combinations on the evolution of antibiotic resistance in Pseudomonas aeruginosa biofilms. NPJ Biofilms Microbiomes 10:156. doi:10.1038/s41522-024-00640-3 39738092 PMC11685532

[18] Uruén C, Chopo-Escuin G, Tommassen J, Mainar-Jaime RC, Arenas J. 2020. Biofilms as promoters of bacterial antibiotic resistance and tolerance. Antibiotics (Basel) 10:3. doi:10.3390/antibiotics10010003 33374551 PMC7822488

[19] Gaio D, Anantanawat K, To J, Liu M, Monahan L, Darling AE. 2022. Hackflex: low-cost, high-throughput, illumina nextera flex library construction. Microb Genom 8:000744. doi:10.1099/mgen.0.000744 35014949 PMC8914357

[20] Sada M, Kimura H, Nagasawa N, Akagawa M, Okayama K, Shirai T, Sunagawa S, Kimura R, Saraya T, Ishii H, Kurai D, Tsugawa T, Nishina A, Tomita H, Okodo M, Hirai S, Ryo A, Ishioka T, Murakami K. 2022. Molecular evolution of the Pseudomonas aeruginosa DNA Gyrase gyrA gene. Microorganisms 10:1660. doi:10.3390/microorganisms10081660 36014079 PMC9415716

[21] Chen MW, Kotaka M, Vonrhein C, Bricogne G, Rao F, Chuah MLC, Svergun D, Schneider G, Liang Z-X, Lescar J. 2012. Structural insights into the regulatory mechanism of the response regulator RocR from Pseudomonas aeruginosa in cyclic Di-GMP signaling. J Bacteriol 194:4837–4846. doi:10.1128/JB.00560-12 22753070 PMC3430337

[22] Bergeron JRC, Fernández L, Wasney GA, Vuckovic M, Reffuveille F, Hancock REW, Strynadka NCJ. 2016. The structure of a type 3 secretion system (T3SS) ruler protein suggests a molecular mechanism for needle length sensing. J Biol Chem 291:1676–1691. doi:10.1074/jbc.M115.684423 26589798 PMC4722450

[23] Nair HAS, Subramoni S, Poh WH, Hasnuddin NTB, Tay M, Givskov M, Tolker-Nielsen T, Kjelleberg S, McDougald D, Rice SA. 2021. Carbon starvation of Pseudomonas aeruginosa biofilms selects for dispersal insensitive mutants. BMC Microbiol 21:255. doi:10.1186/s12866-021-02318-8 34551714 PMC8459498

[24] Jørgensen KM, Wassermann T, Jensen PØ, Hengzuang W, Molin S, Høiby N, Ciofu O. 2013. Sublethal ciprofloxacin treatment leads to rapid development of high-level ciprofloxacin resistance during long-term experimental evolution of Pseudomonas aeruginosa. Antimicrob Agents Chemother 57:4215–4221. doi:10.1128/AAC.00493-13 23774442 PMC3754285

[25] Qi W, Jonker MJ, de Leeuw W, Brul S, ter Kuile BH. 2023. Reactive oxygen species accelerate de novo acquisition of antibiotic resistance in E. coli. iScience 26:108373. doi:10.1016/j.isci.2023.108373 38025768 PMC10679899

[26] Zabłotni A, Schmidt M, Siwińska M. 2024. The SOS response activation and the risk of antibiotic resistance enhancement in Proteus spp. strains exposed to subinhibitory concentrations of ciprofloxacin. Int J Mol Sci 26:119. doi:10.3390/ijms26010119 39795976 PMC11720175

[27] Chittò M, Berger M, Klotz L, Dobrindt U. 2020. Sub-Inhibitory concentrations of SOS-response inducing antibiotics stimulate integrase expression and excision of pathogenicity islands in uropathogenic Escherichia coli strain 536. Int J Med Microbiol 310:151361. doi:10.1016/j.ijmm.2019.151361 31640923

[28] Pribis JP, Zhai Y, Hastings PJ, Rosenberg SM. 2022. Stress-induced mutagenesis, gambler cells, and stealth targeting antibiotic-induced evolution. mBio 13:e0107422. doi:10.1128/mbio.01074-22 35658528 PMC9239211

