# Supplementary material for: Evolutionary adaptations of Pseudomonas aeruginosa biofilms to ciprofloxacin and antioxidant co-treatment in synthetic sputum medium
Source: Microbiol Spectr. 2026 Feb 12;14(3):e03149-25. doi: 10.1128/spectrum.03149-25 (PMC12955469; doi:10.1128/spectrum.03149-25)
Supplement: Supplemental material — Tables S1 and S2; Fig. S1. [file spectrum.03149-25-s0005.docx]

**Supplementary Data**

| Condition | MIC50 (mg/L) |
| --- | --- |
| C | 0.125 |
| CIP | 1 |
| CIP+ED | 0.25 |
| CIP+NAC | 0.5 |
| CIP+THU | 0.5 |

Supplementary Table 1:MIC50 calculated from broth microdilution assay for the different evolved populations in passage 6.

| Gene ID | Gene name |
| --- | --- |
| **Antibiotic resistance** | |
| PA4600 | *nfxB* |
| **Metabolism** | |
| PA1066 |  |
| PA1377 |  |
| PA3516 |  |
| PA0794 |  |
| PA0196 | *pntB* |
| PA3169 | *mtnA* |
| PA0230 | *pcaB* |
| PA2249 | *bkdB* |
| **Transport** |  |
| PA2099 |  |
| PA2553 |  |
| PA1067 |  |
| **Hypothetical** | |
| PA2296 |  |
| PA2865 |  |
| PA5383 |  |
| PA4508 |  |
| **Signal transduction** | |
| PA5272 | *cyaA* |
| PA0176 | *aer2* |
| PA1798 | *parS* |
| PA3409 | *HasS* |
| **DNA replication** | |
| PA4745 | *nusA* |
| **biofilm formation** | |
| PA4226 | *pchE* |
| PA1695 | *pscP* |

Supplementary Table 2: Mutations exhibited in common genes between the current SCFM2 study and a recent study of murine chronic lung infection (15), in different isolates (see Figure 5B).

**
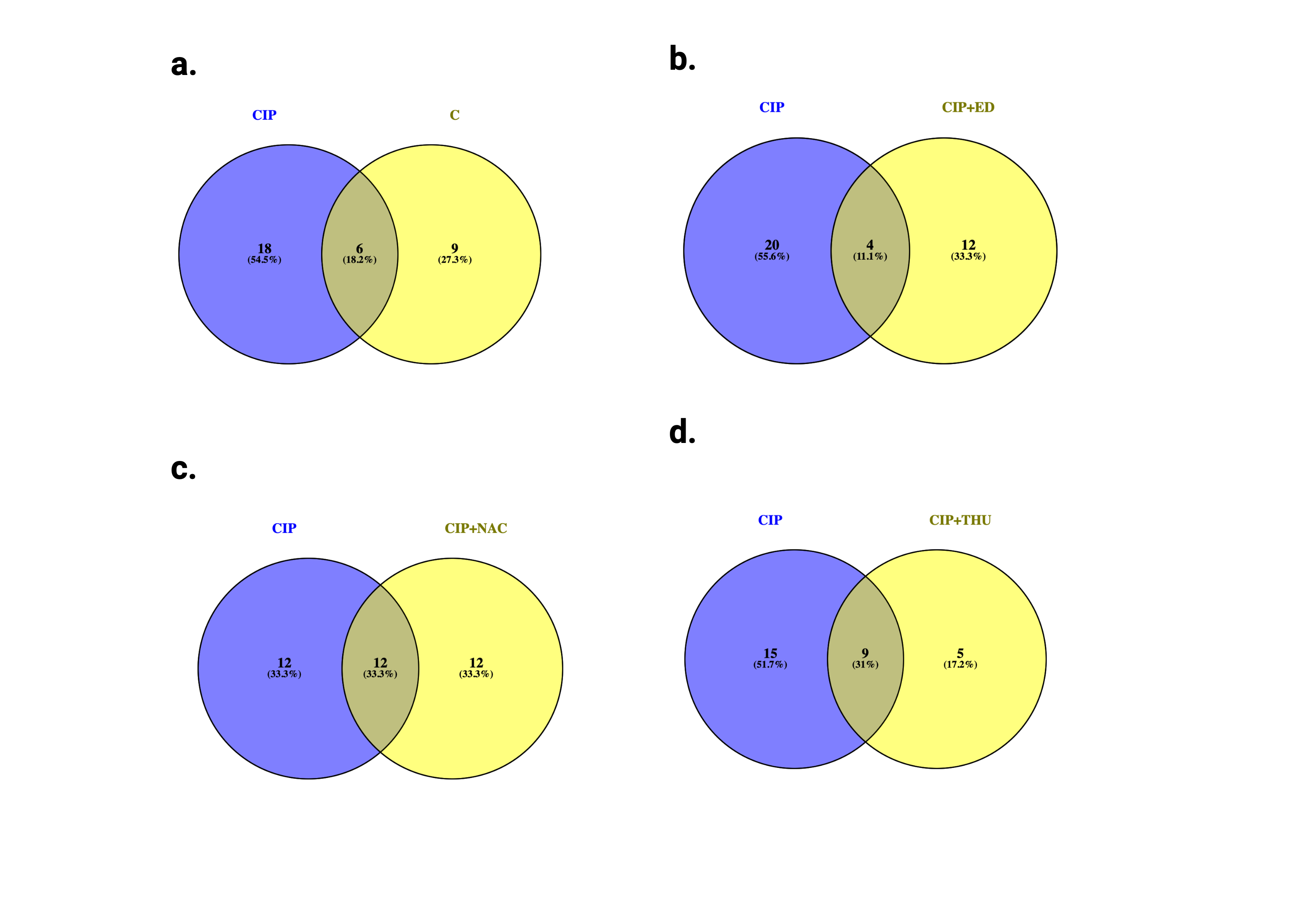
**

Fig. S1: The overlapping regions in the Venn diagrams represent mutated genes shared between treatment conditions. These mutations occur within the same genes, but they may be located at the same nucleotide site or at different nucleotide sites.

**Supplementary File 1.**
List of genes mutated in the four isolates from each condition collected at passage 6 (final passage). Treatment-specific mutations are shown in red, and mutations occurring across multiple conditions are shown in black.

**Supplementary File 2.**
**Sheet 1:** Number of isolates collected at passage 6 (final passage) harboring mutations in each gene for the different conditions (control, CIP, CIP + ED, CIP + NAC, and CIP + THU).
**Sheet 2:** Lists of genes mutated in SCFM2 and in the in vivo study (15), including genes uniquely mutated in SCFM2, genes uniquely mutated in the in vivo study (15), and genes commonly mutated in both SCFM2 and in vivo (15).

**Supplementary File 3.**
List of genes mutated in two evolved bacterial populations from each condition at passage 6 (final passage). Treatment-specific mutations are shown in red, and mutations occurring across multiple conditions are shown in black.

**Supplementary File 4.**
An Excel spreadsheet containing the whole-genome sequencing (WGS) analysis data.
